# DocOx (AIO-PK0106): a phase II trial of docetaxel and oxaliplatin as a second line systemic therapy in patients with advanced pancreatic ductal adenocarcinoma

**DOI:** 10.1186/s12885-016-2052-4

**Published:** 2016-01-15

**Authors:** Thomas J. Ettrich, Lukas Perkhofer, Goetz von Wichert, Thomas M. Gress, Patrick Michl, Holger F. Hebart, Petra Büchner-Steudel, Michael Geissler, Rainer Muche, Bettina Danner, Volker Kächele, Andreas W. Berger, Melanie Güthle, Thomas Seufferlein

**Affiliations:** Department of Internal Medicine I, Ulm University, Albert-Einstein-Allee 23, D-89081 Ulm, Germany; Department of Internal Medicine, Schön-Klinik Hamburg-Eilbeck, Hamburg, Germany; Department of Gastroenterology, Endocrinology, Metabolism and Infectiology, Philipps University of Marburg, Marburg, Germany; Department of Internal Medicine I, Martin-Luther-University, Halle (Saale), Germany; Department of Internal Medicine, Stauferklinikum Schwaebisch-Gmuend, Mutlangen, Germany; Department of Internal Medicine, Oncology/Hematology, Gastroenterology, Esslingen Hospital, Esslingen, Germany; Institute of Epidemiology and Medical Biometry, Ulm University, Ulm, Germany; Praxis für Hämatologie und Onkologie Ulm, Ulm, Germany

**Keywords:** Pancreatic cancer, Advanced disease, Second line therapy

## Abstract

**Background:**

The current study was conducted to examine the activity of a docetaxel/oxaliplatin (DocOx) combination as second line treatment for advanced pancreatic ductal adenocarcinoma (Trial registration: NCT00690300. Registered June 2, 2008)

**Methods:**

DocOx is a prospective, multi-center, single arm, phase II trial using docetaxel (75 mg/m^2^, 60 min, d 1) and oxaliplatin (80 mg/m^2^, 120 min, d 2) in 21-day cycles. The treatment period was scheduled for up to 8 cycles. Primary endpoint was tumor response according to RECIST 1.0. Secondary endpoints were progression free survival, overall survival, safety/toxicity, quality of life and clinical benefit.

**Results:**

Data represent the intention to treat analysis of 44 patients with chemorefractory pancreatic cancer enrolled between 2008 and 2012 at five institutions in Germany. The primary endpoint of tumor response was achieved in 15.9 % of the patients (7 partial remissions, no complete remission), with a disease control rate of 48 % after the first two treatment cycles. Median progression free survival (PFS) was 1.82 months (CI 95 % 1.5–3.96 months) and median overall survival (OS) was 10.1 months (CI 95 % 5.1–14.1 months).

**Conclusions:**

This single-arm trial demonstrates that the combination of docetaxel and oxaliplatin yields promising results for the treatment of advanced pancreatic ductal adenocarcinoma patients. Selected patients had particular benefit from this treatment as indicated by long PFS and OS times. Even after 8 cycles of treatment with DocOx a partial response was observed in 2 patients and stable disease was observed in another 6 patients. The data obtained with the DocOx protocol compare well with other second line protocols such as OFF (oxaliplatin, 5-FU, leucovorin). The DocOx regimen could be an interesting option for patients who received gemcitabine as first line treatment for metastatic pancreatic cancer.

**Electronic supplementary material:**

The online version of this article (doi:10.1186/s12885-016-2052-4) contains supplementary material, which is available to authorized users.

## Background

Pancreatic ductal adenocarcinoma (PDAC) is a major cause of cancer related deaths in the Western world. The only curative option for PDAC is surgery, but at the time of primary diagnosis only 10–15 % of patients are eligible for surgery with curative intent. The main limitation is the delayed diagnosis at an already locally advanced or metastatic state of the disease [1, 2]. Consequently, systemic therapy is the treatment of choice for the majority of patients. The standard of care in this setting has developed over the last decade. FOLFIRINOX and the combination of gemcitabine/nab-paclitaxel have proven to be superior to single agent gemcitabine in the first-line therapy of metastatic PDAC [3, 4]. Second line strategies in PDAC achieve a median progression free survival (mPFS) of 4 months and a median overall survival (mOS) of 6 months, respectively [5]. However, the optimal second-line strategy for PDAC still remains to be defined [6, 7]. Compared to best supportive care (BSC) or 5-fluorouracil (5-FU) alone the combination of 5-FU, leucovorin and oxaliplatin (OFF) significantly prolonged the overall survival time in ECOG 0–2 (Eastern Cooperative Oncology Group) patients [8, 9]. Recently, the combination of nanoliposomal irinotecan plus 5-FU has also shown superiority as second line treatment for PDAC compared to 5-FU alone (mPFS 3.1 versus 1.5 months, HR 0.56; mOS 6.1 versus 4.2 months, HR 0.67) [10]. The objective response rate is generally low in the second line setting [11]. Single agent docetaxel achieves response rates of up to 15 % as first line therapy of advanced PDAC [12, 13], and has moderate activity as second line treatment of PDAC in retrospective analyses [14, 15]. Oxaliplatin-based combination regimen show similar response rates as docetaxel [16–18]. Several phase I/II studies confirmed the efficacy and safety of the combination of docetaxel plus oxaliplatin for different tumor entities [19–21]. To date the combination of both substances has not been evaluated in the treatment of chemorefractory PDAC. The current study was conducted to prospectively evaluate the activity and feasibility of the combination of docetaxel/oxaliplatin (DocOx) as second line treatment of PDAC.

**Fig. 1:**
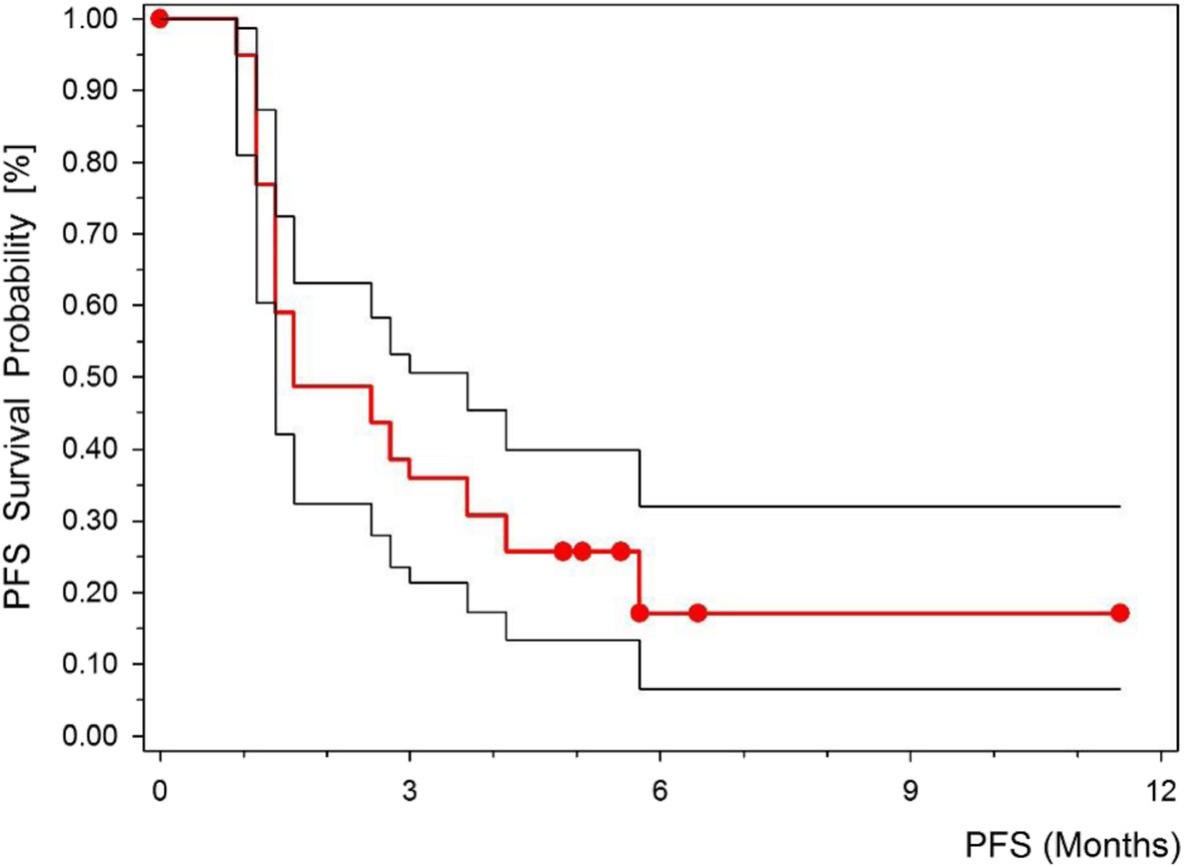
Kaplan-Meier plot: second line progression free survival time with 95 % confidence interval. *PFS* progression free survival

**Fig. 2:**
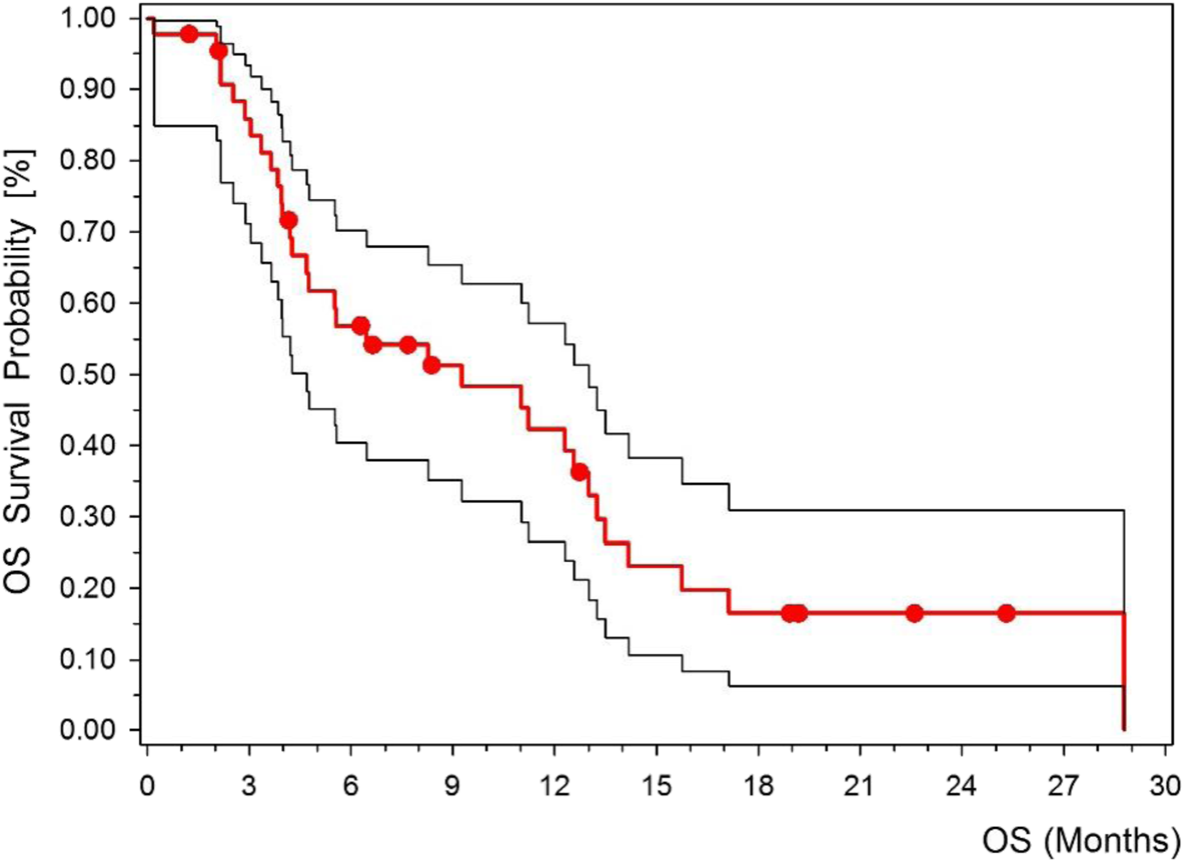
Kaplan-Meier plot: second line overall survival time with 95 % confidence interval. *OS* overall survival

## Patients and methods

The DocOx trial (NCT00690300) was designed as an open label, multicenter, single arm, phase II study. Between February 2008 and March 2012, 47 patients were enrolled at five German institutions. The final analysis was restricted to 44 patients.

### Patient population

Inclusion criteria were as follows: histologically or cytologically confirmed metastatic or unresectable locally advanced PDAC; age ≥18 years; at least one measurable target lesion according to RECIST 1.0 (Response Evaluation Criteria in Solid Tumors) outside any previously irradiated area; failure of first line therapy of metastatic or unresectable locally advanced PDAC due to progressive disease during or within 3 months after finishing first line chemotherapy; Karnofsky performance score (KPS) >60 % (ECOG 0–2); life expectancy ≥12 weeks; adequate bone marrow function (granulocyte count ≥1.5x10^9^/L, platelet count ≥100x10^9^/L, hemoglobin ≥9 g/dl); serum bilirubin levels <2 times upper limit of normal (ULN), up to 2.5 times ULN in case of hepatic metastasis (biliary drainage allowed); transaminases <2.5 times ULN.

Exclusion criteria were as follows: Any other primary tumor or secondary malignancy except basal cell carcinoma of the skin or in situ carcinoma of the cervix uteri (patients with adequately treated other malignancies and tumor absence for ≥5 years were eligible); pregnancy or breastfeeding period; patients unable to ensure adequate contraception; known cerebral metastasis; uncontrolled severe infections; peripheral neuropathy exceeding CTCAE (Common Terminology Criteria for Adverse Events) grade 1.

All patients signed a written informed consent according to national and local regulations. The protocol was approved by the Ethics Committee of Ulm University [22].

### Treatment plan

In this open label trial patients received docetaxel 75 mg/m^2^ (60 min iv infusion) on day 1 and oxaliplatin 80 mg/m^2^ (120 min iv infusion) on day 2, repeated every 3 weeks. Treatment was administered at least for 8 cycles, unless there was tumor progression, unacceptable toxicity or patient refusal. In case of stable disease (SD) after 8 cycles patients could choose to carry on with the therapy. Premedication included adequate antiemetic therapy and oral dexamethasone 8 mg the day prior to docetaxel application as well as on days 2 and 3 after docetaxel treatment. On treatment days the patients received another 16 mg of dexamethasone iv. To prevent oxaliplatin related polyneuropathy 1 g calcium gluconate and 1 g magnesium gluconate were administered iv prior to and after oxaliplatin infusion. In case of severe hematotoxicity prophylactic granulocyte colony-stimulating factor treatment in addition to dose modification was applied. In case of neutropenia <1.5x10^9^/L, thrombopenia <100x10^9^/L, diarrhea > grade 1, peripheral neuropathy > grade 1 or other nonhematologic toxicities > grade 1 treatment could be delayed up to a maximum of 2 weeks. In case of a 2 weeks delay and ongoing neutropenia the dose of both cytostatics was reduced to 50 % if neutrophiles were 1.0 to 1.5x10^9^/L or if platelets were 50 to 100x10^9^/L. Treatment was discontinued in case of neutropenia <1.0x10^9^/L, thrombopenia <50x10^9^/L, peripheral neuropathy grade 2 or other ongoing non-hematologic toxicities after 2 weeks delay. The dose of both drugs was reduced to 80 % if granulocytes were less than <0.5x10^9^/L or <1.0x10^9^/L with fever exceeding 38.5 °C, if platelets were <50x10^9^/L or in case of non-hematologic toxicity > grade 2 (except alopecia, nausea and vomiting). In case of peripheral neuropathy grade 2 oxaliplatin was reduced to 50 % and docetaxel to 80 % of the previous dose level after recovery. Toxicity was assessed using the National Cancer Institute (NCI) common toxicity criteria (CTC) version 3. Repeated severe toxicity after the second dose adjustment resulted in termination of the treatment.

### Pretreatment evaluation and follow-up

The baseline evaluation included a complete medical history, physical examination including vital signs, an electrocardiogram (ECG), complete blood count (CBC) plus serum chemistry and, in case of child-bearing age, a pregnancy test, all within one week prior to start of treatment. A chest x-ray and abdominal computed tomography were required to define the target lesion(s). All patients received questionnaires to assess quality of life (EORTC QLQ-C30) and clinical benefit (pain, use of analgesics, body weight, Karnofsky performance score) prior to each treatment cycle, furthermore the clinical benefit was recorded weekly within each cycle. Assessments before start and then weekly within each cycle included physical examination, CBC plus serum chemistry and recording of adverse events. Tumor response was evaluated by computed tomography after every second cycle according to RECIST 1.0 for the defined target lesions. Planned study termination after 8 cycles was followed by six- weekly examinations including assessment of life status, physical examination and Karnofsky performance score. The treatment was stopped in case of progressive disease, inacceptable toxicity, incompliance, or patient’s wish.

### Treatment evaluation

The primary endpoint of the study was defined as tumor response according to RECIST 1.0. Toxicities were graded according to NCI CTC version 3.0 Severe adverse events (SAE) were defined as follows: any reaction, side effect or disease displaying an increased risk or danger for the patient. Quality of life was assessed based on the EORTC QLQ-C30. Moreover, clinical benefit (CB) was recorded in all patients (Additional file 1).

### Statistical analysis

Tumor response was defined as the primary endpoint of the study. Secondary endpoints included PFS, OS, quality of life and clinical benefit. All patients treated for at least one cycle of chemotherapy, even in case of protocol violation, were included into final analysis on an intention to treat (ITT) basis. The trial was based on a Simon’s two-stage design [23]. For the sample size calculation a response rate of ≥15 % was considered sufficient in an interim analysis, whereas a rate ≤5 % was insufficient. The size of the type I (α) and II (β) errors were 0.1 and 0.2, respectively. An interim analysis was planned after 22 patients and in case of no response the study would be closed prematurely. Otherwise another 22 patients were to be enrolled until the total number of 44 participants was reached. OS and PFS were estimated using the Kaplan-Meier method. A descriptive data analysis was done for EORTC-QLQ-C30 and clinical benefit. The clinical benefit was calculated from four parameters (pain-intensity, use of pain-medication, KPS and body-weight) [24]. Pain intensity/use of analgetics and KPS were defined as primary indicators, body weight counted as a secondary indicator. For the evaluation of the clinical benefit patients had either to be positive, stable or negative classified for the primary indicators. Only in case of stable primary indicators the secondary indicator, body weight, was included for overall assessment. For positive evaluation of the clinical benefit at least 4 weeks of improvement of the indicators were required. A diagram for the assessment of the clinical benefit is displayed in the (Additional file 2).

## Results

### Patients characteristics

A total of 47 patients were recruited between February 2008 and March 2012 at five German institutions. The primary analysis was restricted to 44 patients (ITT-population). Three patients did not start treatment due to death (two patients) or refusal (one patient). The first stage of the study included 22 patients for interim analysis. After fulfilling the preset requirements for proceeding of the trial (response rate ≥15 %) another 22 patients were enrolled. The patient characteristics are listed in Table 1. As first line treatment all patients received a gemcitabine-based regimen, except two patients who received a 5-FU based concept. The median duration of first line therapy was 4.5 months (2.1–7.25 months). The main reasons for discontinuation of first line therapy were progressive disease in 42 patients (95.5 %) and toxicity in two cases (4.5 %). Most of the patients (81.8 %, 36/44) had metastatic disease at initiation of second line therapy.

**Table 1:** Baseline Characteristics

Docetaxel/Oxaliplatin (*n* = 44)		
Patient Characteristics	Number of Patients	Percent
Sex		
Male	29	65.9
Female	15	34.1
Age (years)		
Median (and range)	66.5	38–76
Karnofsky performance status score (*n* = 43)		
100 %	9	20.9
90 %	21	48.8
80 %	13	30.2
Prior surgery		
No	26	59
Curative Intention	9	20.5
Palliative	9	20.5
Prior radiotherapy		
Yes	3	6.8
No	41	93.2
Location of the primary		
Head	26	59.1
Body	10	22.7
Tail	8	18.2
Disease extension		
Locally advanced	8	18.2
Metastatic	36	81.8
Metastatic sites^a^		
Liver	30	68.1
Lymphnodes	10	22.7
Lung	7	15.9
Bone	2	4.5
Median duration of first line therapy (mts)	4.5	95 % CI 2.1–7.25

^a^multiple presentations included; *95 % CI* confidence interval

### Dose intensity and efficacy

In median 3 weeks passed between termination of the first line therapy and start of the second line treatment. From the ITT-population four patients (9.1 %) received only 1 cycle, another twelve (27.3 %) only 2 cycles, respectively. The median number of chemotherapy cycles was 4 (range: 1–8 cycles). Nine patients (20.5 %) completed the pre-planned 8 cycles of chemotherapy. Ten patients were eligible for final staging by computed tomography after 8 cycles: two had continuous partial response (PR), six stable disease (SD) and two progressive disease (PD). The final analysis includes one patient with a total of 7 cycles chemotherapy who refused the last cycle. Interestingly, even after 8 cycles of treatment with DocOx, a partial response was observed in two patients and stable disease in another six patients corresponding a disease control rate of 18 %.

The main reason for discontinuation of treatment was PD in 28 cases (63.6 %) Toxicity and death were in charge for therapy discontinuation each within three cases (6.8 %). Seven patients (15.9 %) completed the planned treatment of 8 cycles and four continued therapy beyond the planned treatment period. The median relative dose intensity for both drugs was 95.7 % of the theoretical dose for the applied cycles. The dose intensity was slightly higher for docetaxel compared to oxaliplatin (97.5 % vs. 93.5 %).

All patients had at least one measurable lesion for response assessment. Seven patients exhibited a partial response according to RECIST 1.0 (15.9 %, 95 % CI 10–26 %, see Table 2). Stable disease was observed in 14 patients (31.8 %). The calculated disease control rate (DCR) was 47.7 %. The DCR was defined as the proportion of patients with PR or SD for at least 2 cycles. The median PFS was 1.82 months (CI 95 % 1.5–3.96 months), Fig. 1. The PFS rate at 6 months and 1 year was 17.1 % in both cases. The median OS was 10.1 months (CI 95 % 5.1–14.1 months), Fig. 2. OS rates were 56.8 % at 6 months and 39.3 % at 1 year, respectively. There is one exceptional long time survivor with an overall survival of 75 months from primary diagnosis and a PFS of 36 months after start of second line treatment with a total of 22 cycles of docetaxel/oxaliplatin.

**Table 2:** Response and Survival

Docetaxel/Oxaliplatin (*n* = 44)		
Efficacy	Number of Patients	Percent
Response		
Complete response (CR)	0	
Partial response (PR)	7	15.9
Stable disease (SD)	14	31.8
Progressive disease (PD)	23	52.3
Disease control rate (CR + PR + SD)	21	47.7
Survival	months	95 % CI
Median progression free survival	1.82	1.5–3.96
Median overall survival	10.1	5.1–14.1

*95 % CI* confidence interval

### Quality of life and clinical benefit

Quality of life (QoL) was assessed using the QLQ-C30 questionnaire. A descriptive analysis revealed relevant changes in quality of life. Interestingly, QoL was independent from therapy response. The clinical benefit was calculated from the four parameters pain-intensity, use of analgesics, KPS and body-weight according to Burris et al. [24] and as described above. Five patients (11.4 %) described a clinical benefit (two patients with a tumor response and three without response) at different time points of study termination. In 39 cases (88.6 %) the score worsened. The CB parameters are listed in Table 3. Pain intensity was stable or decreased in the majority of patients (90.2 %, 37/41) throughout the therapy. The KPS was stable in 28 patients (63.6 %) during the course of treatment. Any loss of body weight was noticed in 40 patients (90.9 %).

**Table 3:** Clinical benefit

Docetaxel/Oxaliplatin (*n* = 44)			
	Number of Patients	Percent	95 % CI
Clinical benefit response	5	11.4	3.79–24.56
Pain intensity (*n* = 41)			
Decreased	4	9.8	2.72–23.13
Stable	29	70.7	54.46–83.87
Improved	8	19.5	8.82–34.87
Karnofsky Perfomance Score			
Decreased	16	36.4	22.41–52.23
Stable	28	63.6	47.77–77.59
Body weight			
Decreased	40	90.9	78.33–97.47
Increased	4	9.1	2.53–21.67

*95 % CI* confidence interval

From the collected data for Global Health Status out of the EORTC QLQ-C30 questionnaire we calculated the median time until definitive deterioration (TUDD). The TUDD was calculated in accordance to the published papers of Anota et al. and Bonnetain et al. and defined as an ongoing deterioration of at least five points as compared to the baseline [25, 26]. The median TUDD was calculated with 3.5 months.

### Safety

Treatment had to be discontinued in three patients (6.8 %) due to hematologic toxicity. Table 4 summarizes the most frequent adverse events. Neutropenia grade 3 to 4 occurred in 63.6 % (28/44) of patients. Febrile neutropenia grade 3 to 4 was reported in 4.5 % (2/44) of patients. However, only two patients (4.5 %) required granulocyte-colony stimulating factor (G-CSF) at least once during treatment. The major non-hematologic grade 3 or 4 adverse events were diarrhea (11.4 %, 5/44) and nausea (9.1 %, 4/44). Grade 1 or 2 peripheral neuropathy was reported in 52.3 % (23/44) of patients, ≥grade 3 in only one patient (2.3 %). The most common grade 1 or −2 toxicities were alopecia (68.2 %, 30/44) and mucositis (29.3 %, 13/44). A more detailed overview of the toxicities is shown in the (Additional file 1: Table S1). No unexpected toxicities were reported.

**Table 4:** Common Grade 3 or 4 Adverse Events

Docetaxel/Oxaliplatin (*n* = 44)		
Adverse Event	Number of Patients	Percent
Hematologic		
Neutropenia	28	63.6
Febrile Neutropenia	2	4.5
Thrombocytopenia	1	2.3
Anaemia	1	2.3
Non Hematologic		
Fatigue	2	4.5
Diarrhea	5	11.4
Nausea	4	9.1
Peripheral Neuropathy	1	2.3
G-CSF use	2	4.5

*G-CSF* granulocyte-colony stimulating factor

## Discussion and conclusions

An increasing number of patients with PDAC are eligible for a second line therapy. A recently published systematic review found beneficial effects for second line chemotherapies compared to best supportive care in PDAC, in particular for combinations of platinum agents and fluorouracil or gemcitabine [5]. However, there is currently no standard of care in the second line setting in PDAC. Evidence is mainly based on few small phase II trials and one phase III trial [8, 9, 18, 27, 28]. The CONKO-003 trial demonstrated that the combination of oxaliplatin, 5-FU and leucovorin according to the OFF regimen extends the duration of overall survival compared to 5-FU alone or to best supportive care [8, 9].

This phase II trial was conducted to establish the efficacy and safety of the combination of docetaxel and oxaliplatin in the second line setting and its impact on the quality of life and clinical benefit for patients with advanced, chemorefractory PDAC. Both substances have shown interesting response rates in the first [12, 13] and second line [14, 15] setting in metastatic PDAC as single agents or in combination. Being aware of the limitations of a single arm trial the combination of docetaxel/oxaliplatin achieved a response rate of 15.9 %, a DCR of 47.7 %, a median OS of 10.1 months (CI 95 % 5.1–14.1 months) and a median PFS of 1.82 months (CI 95 % 1.5–3.96 months). These data are comparable to other published protocols such as OFF (see Table 5). Interestingly, even after 8 cycles of treatment with DocOx, a partial response was observed in two patients and stable disease in another six patients corresponding to a disease control rate of 18 %. There is one exceptional long time survivor with an overall survival of 75 months from primary diagnosis and a PFS of 36 months after start of second line treatment with a total of 22 cycles of docetaxel/oxaliplatin that is still alive.

**Table 5:** Second line therapies in advanced PDAC

Regimens	Phase	Patients	mPFS (ms)	mOS (ms)	OS 6-ms %	DCR %	PR %	Ref
FOLFIRINOX	Ret	22	5.4	8.5	n.a.	63	19	[32] 2011
FOLFIRINOX	Ret	18	2.8	8.4	44.4	55.6	27.8	[33] 2013
Nab-Paclitaxel	II	19	1.7	7.3	58	37	5	[31] 2012
FOLFIRI	II	50	3.2	5.0	32	36	8	[18] 2012
Capecitabine/Docetaxel	II	43	3.7	5.3	n.a.	73	14	[34] 2014
mFOLFOX	II	30	1.5	3.7	30	17	7	[28] 2009
FOLFIRI		31	2.1	4.2	27	23	0	
OFF	III	23	n.a.	4.8	n.a.	n.a.	n.a.	[8] 2011
BSC		23		2.3				
OFF	III	76	2.9	5.9	n.a.	n.a.	n.a.	[9] 2014
FF		84	2.0	3.3				
Xelox	II	39	2.5	5.8	44	28.6	2.6	[35] 2008
Docetaxel	Ret	17	2	4	n.a.	35	6	[15] 2010
Nal-Iri	II	40	2.4	5.2	42.5	50	7.5	[27] 2013
Nal-Iri/5-FU/LV	III	417	3.1	6.1	n.a.	n.a.	n.a.	[10] 2014
5-FU/LV			1.5	4.2				
GEMOX	II	33	4.2	6	n.a.	61.3	22.6	[36] 2006
Vatalanib	II	67	2	n.a.	29	31	3	[37] 2014
DocOx	II	44	1.8	10.1	56.8	48	15.9	

*mPFS* median progression free survival time, *mOS* median overall survival time, *Ref* reference, *Ret* Retrospective, *ms* months, *DCR* disease control rate, *PR* partial remission, *ms* months, *OFF* oxaliplatin, folinic acid, fluorouracil, *LV* leucovorine, 5-FU 5-fluorouracil, *Nab-Paclitaxel* nanoalbumine bound pacitaxel, *Nal-Iri* nanoliposomal irinotecan, *FOLFIRI* fluorouracil, leucovorine, irinotecan, *FOLFOX* fluorouracil, leucovorine, oxaliplatin, *FOLFIRINOX* fluorouracil, leucovorine, irinotecan, oxaliplatin, *DocOx* docetaxel oxaliplatin, *n.a.* not applicable, *Ref* Reference and publication date

In our study the combination of docetaxel/oxaliplatin was in general well tolerated and no unexpected toxicities occurred during therapy. Most subjects experienced at least one grade 3/4 adverse event, mainly hematological (neutropenia, 63.6 %) and diarrhea (11.4 %) see Table 4. Compared to the OFF regimen [8, 9], the DocOx protocol appears to be more toxic. However, all toxicities were manageable. 15 patients (34.1 %) were still eligible to third line therapy after progress to DocOx, see Table 6. The rate of febrile neutropenia was low (<5 %) and only two patients required single doses of G-CSF. A calcium and magnesium infusion was applied at each cycle because at the time the trial was conducted this was supposed to prevent oxaliplatin-associated polyneuropathy [29]. However, new data refute this concept [30].

**Table 6:** Third line therapies after failure of Docetaxel/Oxaliplatin treatment

Third line therapy (*n* = 15)		
Treatment	Number of Patients	Percent
Gemcitabine + Capecitabine	8	53.3
5-FU/Oxaliplatin based	3	20
5-FU/Irinotecan based	4	26.7

5-FU/Oxaliplatin based (OFF, FUFOX, XELOX, FOLFOX); 5-FU/Irinotecan based (XELIRI, FOLFIRI)

There are few data on quality of life and clinical benefit during second line treatment of patients with PDAC. For the assessment of clinical benefit we used a composite score of pain and analgesics requirements, Karnofsky performance status, and body weight. In total five patients (11.4 %) reported a clinical improvement at final examination. Cross trial comparisons to other second line trials are difficult, due to different chemotherapy regimens, heterogeneous patient collectives, distinct definitions of clinical benefit and mostly due to the fact that only few data are available. Recently, comparable results with an improvement of clinical benefit in 20 % of patients were reported in a phase II trial using nanoliposomal-irinotecan as a single agent [27]. The median time until definitive deterioration of the Global health status was calculated with 3.5 months and is comparable to data from first line settings published so far [25].

The major limitation of our data is the single arm design. However, there was no established second line chemotherapy available when this trial was initiated. Furthermore, similar trials confirmed that patients eligible for a second line treatment do not agree to be randomized to best supportive care only [8]. The data of this study compare well with those obtained with other protocols including the OFF regimen (see Table 5) and make this combination an option for patients with chemorefractory PDAC.

A second limitation is the fact that by now a substantial number of patients will have received oxaliplatin or a nab-paclitaxel in the first line setting due to the increased use of FOLFIRINOX or the gemcitabine/nab-paclitaxel regimen. However, a significant number of patients will still receive gemcitabine +/− erlotinib in the first line setting and could benefit from docetaxel/oxaliplatin as second line treatment. Moreover, patients with an early relapse after adjuvant gemcitabine therapy who are not eligible for FOLFIRINOX maybe candidates for DocOx. In particular, the acceptable safety profile and the promising data on efficacy, quality of life and clinical benefit make this combination an interesting option for patients with chemorefratory pancreatic cancer. Recently, single agent nab-paclitaxel (phase II) [31] and the combination of nano-liposomal irinotecan/ 5-FU/ LV (phase III) [10] demonstrated promising results in this setting. It remains to be elucidated whether modern formulations of taxanes or irinotecan are superior to docetaxel in this setting.

## Additional files

Additional file 1: Table S1. Adverse Events independent from relation to therapy. (DOCX 26.2 kb) Additional file 2: Figure S1. Consort diagram. **Figure S2.** Flow chart for the assessment of the clinical benefit. (DOCX 82.8 kb)

## Abbreviations

BSC: best supportive care
CB: clinical benefit
CBC: complete blood count
CI: confidence interval
CTC: common toxicity criteria
CTCAE: Common Terminology Criteria for Adverse Events
DCR: disease control rate
DocOx: docetaxel/oxaliplatin
ECG: electrocardiogram
ECOG: Eastern Cooperative Oncology Group
EORTC: European Organization for Research and Treatment of Cancer
5-FU: 5-fluorouracil
FOLFIRINOX: fluorouracil leucovorine, irinotecan, oxaliplatin
G-CSF: granulocyte-colony stimulating factor
HR: hazard ratio
ITT: intention to treat
KPS: karnofsky performance score
(m)OS: (median) overall survival
(m)PFS: (median) progression free survival
NCI: National Cancer Institute
OFF: 5-FU leucovorin, oxaliplatin
PD: progressive disease
PDAC: pancreatic ductal adenocarcinoma
PR: partial response
QLQ-C30: quality of life questionnaire-core 30
QoL: quality of life
RECIST: Response Evaluation Criteria in Solid Tumors
SAE: severe adverse events
SD: stable disease
TUDD: time until definitive deterioration
ULN: upper limit of normal
