# Supplementary material for: DocOx (AIO-PK0106): a phase II trial of docetaxel and oxaliplatin as a second line systemic therapy in patients with advanced pancreatic ductal adenocarcinoma
Source: BMC Cancer. 2016 Jan 15;16:21. doi: 10.1186/s12885-016-2052-4 (PMC4714522; doi:10.1186/s12885-016-2052-4)
Supplement: Additional file 1: Table S1. — Adverse Events independent from relation to therapy. (DOCX 26.2 kb) [file 12885_2016_2052_MOESM1_ESM.docx]

**Tables (supplementary)**

| **Table 7.** Adverse Events independent from relation to therapy | | |
| --- | --- | --- |
|  | | |
| Docetaxel/ Oxaliplatin  (n = 44) | | |
| Adverse Event | Number of Patients | % |
| CTCAE grade III-IV |  |  |
| Neutropenia | 28/44 | 63.6 |
| Febrile Neutropenia | 2/44 | 4.6 |
| Thrombocytopenia | 1/44 | 2.3 |
| Anaemia | 1/44 | 2.3 |
| Lymphopenia | 5/44 | 11.4 |
| Fatigue | 2/44 | 4.6 |
| Diarrhea | 5/44 | 11.4 |
| Nausea | 4/44 | 9.1 |
| Peripheral Neuropathy | 1/44 | 2.3 |
| Pain | 4/44 | 9.1 |
| Thrombosis/ Embolism | 3/44 | 6.8 |
| Mucositis | 1/44 | 2.3 |
| Constipation | 2/44 | 4.6 |
| CTCAE grade I-II |  |  |
| Peripheral Neuropathy | 23/44 | 52.3 |
| Hair loss/ alopecia | 30/44 | 68.2 |
| Hand-foot skin reaction | 8/44 | 18.2 |
| Nausea | 24/44 | 54.6 |
| Vomiting | 12/44 | 31.8 |
| Constipation | 7/44 | 15.9 |
| Mucositis | 13/44 | 29.6 |
| Pain | 30/44 | 68.2 |
| Thrombosis/ Embolism | 4/44 | 9.1 |
| Lack of appetite | 5/44 | 11.4 |
| Rigors | 3/44 | 6.8 |
| Edema | 13/44 | 29.6 |
