# Supplementary material for: DocOx (AIO-PK0106): a phase II trial of docetaxel and oxaliplatin as a second line systemic therapy in patients with advanced pancreatic ductal adenocarcinoma
Source: BMC Cancer. 2016 Jan 15;16:21. doi: 10.1186/s12885-016-2052-4 (PMC4714522; doi:10.1186/s12885-016-2052-4)
Supplement: Additional file 2: Figure S1. — Consort diagram. Figure S2. Flow chart for the assessment of the clinical benefit. (DOCX 82.8 kb) [file 12885_2016_2052_MOESM2_ESM.docx]

**Figure 3.** Consort diagram

*DocOx docetaxel/oxaliplatin*

**Figure 4.** Flow chart for the assessment of the clinical benefit

^1^ Improvement of both highlighted parameters or improvement of 1 and stabilization of the other

^2^  Both parameters are stable

^3^ Deterioration of at least one of the highlighted parameter

Use of pain medication

Pain intensity

**Pain** medication

**KPS** medication

positive^1^

negative^3^ ^1^

stable^2^

weight

Body weight gain ≥7%

Body weight loss ≤7%
